# Is perception of quality more important than technical quality in patient video cases?

**DOI:** 10.1186/s12909-015-0419-x

**Published:** 2015-08-13

**Authors:** Damian Roland, David Matheson, Nick Taub, Tim Coats, Monica Lakhanpaul

**Affiliations:** 1 Department of Health Sciences, SAPPHIRE Group, University of Leicester, 22-28 Princess Road West, Leicester, LE1 6TP UK; 2 Paediatric Emergency Medicine Leicester Academic Group, University Hospitals of Leicester NHS Trust, Leicester Royal Infirmary, Leicester, LE1 5WW UK; 3 Carnegie Faculty, Leeds Beckett University, Leeds, LS1 3HE UK; 4 Emergency Medicine Academic Group. Cardiovascular Sciences, Leicester University, Leicester, LE3 9QP UK; 5 Child and Adolescent Mental Health, Palliative Care and Paediatrics, Institute of Child Health, London, UK

## Abstract

**Background:**

The use of video cases to demonstrate key signs and symptoms in patients (patient video cases or PVCs) is a rapidly expanding field. The aims of this study were to evaluate whether the technical quality, or judgement of quality, of a video clip influences a paediatrician’s judgment on acuity of the case and assess the relationship between perception of quality and the technical quality of a selection of video clips.

**Methods:**

Participants (12 senior consultant paediatricians attending an examination workshop) individually categorised 28 PVCs into one of 3 possible acuities and then described the quality of the image seen. The PVCs had been converted into four different technical qualities (differing bit rates ranging from excellent to low quality).

**Results:**

Participants’ assessment of quality and the actual industry standard of the PVC were independent (333 distinct observations, spearmans rho = 0.0410, *p* = 0.4564). Agreement between actual acuity and participants’ judgement was generally good at higher acuities but moderate at medium/low acuities of illness (overall correlation 0.664). Perception of the quality of the clip was related to correct assignment of acuity regardless of the technical quality of the clip (number of obs = 330, *z* = 2.07, *p* = 0.038).

**Conclusions:**

It is important to benchmark PVCs prior to use in learning resources as experts may not agree on the information within, or quality of, the clip. It appears, although PVCs may be beneficial in a pedagogical context, the perception of quality of clip may be an important determinant of an expert’s decision making.

## Background

The potential benefits of patient video cases (PVCs) are being increasingly realised [1], with a survey of children’s hospitals in North America and the UK finding that video recordings of clinical interactions and patient signs are relatively common [2]. Video can be a powerful tool as the addition of video to audio clips have been demonstrated to have large effects on the recall of the content of the cases, both objectively and subjectively [3]. The knowledge and learning obtained from PVCs is dependent on a number of factors that have yet to be determined. Information content, technical quality, monitor fidelity, bandwidth availability, processing speed (if digital recording) and interference from other electronic devices all may influence learning from Patient Video Cases [4].

Quality issues are clearly relevant to the validity of assessments of learning outcomes however there is no universal definition of what constitutes high quality video. In a medical context most investigation of video quality has been in relation to telemedicine. The focus of this research has been either in the transfer of single pictures (such as in tele-dermatology [5]), communication between healthcare professionals and patients separated by large geographic distances [6] or specific radiological examinations such as echocardiograms [7]. The endpoint of these studies being a comparison between different clinicians or clinical outcomes of patients, with no examination of the effects of image quality. Literature in the area of image quality and decision making is scant. The Royal College of Paediatrics and Child Health have utilised video cases in their postgraduate examinations since 2004. A review of the process found this particular assessment to be as reliable and informative as more traditional examination methodologies [8]. In 2005 McFaul [9] conducted a feasibility study to test whether real time video pictures used in a clinical environment could be transmitted within a hospital to enable a specialist to form a useful assessment of the severity and nature of a child’s illness. In approximately 70 % of cases the image was felt to be good enough to guide clinical management, although there was variation between particular conditions.

The aim of this study was to assess paediatricians’ perceptions of video quality and acuity of illness in a selection of PVCs of unwell children. The null hypothesis being the technical quality of a video case of an acutely unwell child does not influence a paediatrician’s judgement on the child’s acuity.

The objectives of the study were to:

1. Assess the relationship between senior paediatricians’ perception of quality and the measured technical quality of a selection of video cases.
2. Define the correlation between the paediatricians’ judgments on the acuity of patients clinical signs via video cases and the actual patient acuity
3. Evaluate whether the technical quality, or judgement of quality, of a video case influences the paediatricians judgment on acuity.

## Methods

The video cases used in this study were taken from the “Spotting the Sick Child” educational project [10] and additional footage for which consent had been obtained from parents to demonstrate to health care professionals (The REMIT Study [ISRCTN94772165] which had been given ethical approval by East Midlands Research Ethics Committee 2). This study involved solely health care professionals themselves and is currently exempt from formal ethical review under current United Kingdom national guidance. Clips were converted into four different image quality standards by a multimedia company experienced with dealing with medical images. The four image qualities were based on bitrates of the files. Bitrates are the number of “bits per second” at which the data in a video is being delivered. A higher bitrate means that the video has more information. The display size of the files was kept similar i.e., the dimensional size in centimetres of the image was not bigger for the higher bit-rates. The highest quality was labelled as ‘Excellent’ and was in Mbps (megabits per second). This is of similar quality to a high definition Digital Video Disc (DVD). The other qualities high, medium and low were in Kbps (kilobits per second) with high being of standard definition television quality (above 2500 kbps) and low being similar to quality in a video-conference (below 2000 kbps). Medium sat somewhere in between. The low quality was not so poor as to have been rejected as unrecognisable by a lay person. Participants (12 paediatricians attending an exam standard setting workshop) saw 28 clips at one of four image qualities, with all qualities being seen at least once for each clip across all four groups (a cross-over design to avoid confounding results by repeated clip viewing). The average experience of the paediatricians as a consultant was 16.9 years (range 1–18). One clip was viewed in all 4 bitrates by all participants as a control. The participants were split into four groups of three. This was a pragmatic decision to optimise the number of different video cases seen at the four different video qualities. The paediatricians volunteered themselves to be part of the study which was open to all those who attended the workshop (approximately 40 participants). An example of schemata demonstrating the details of the clips and the order seen is shown in Table 1.

**Table 1:** Schemata for viewing videos (each group contained 3 paediatricians)

Clip	Group A	Group B	Group C	Group D
1	Respiratory low quality	Respiratory medium quality	Respiratory high quality	Respiratory excellent quality
2	Hydration medium quality	Hydration high quality	Hydration excellent quality	Hydration low quality
3	Response to social cues high quality	Response to social cues excellent quality	Response to social cues low quality	Response to social cues medium quality
x…	The four qualities repeated through the five patient categories (Respiratory hydration, Response to social cues, State variation and Colour)			
28	Colour excellent quality	Colour low quality	Colour medium quality	Colour high quality

Participants were asked, for each clip, to assess a range of clinical relevant features across 5 categories [Colour, Response to Social Overture, State Variation, Hydration and Respiratory Effort] from the pro-forma designed by McFaul [9] (Table 2). They then rated the quality of the image seen. There are currently no validated image quality measures specifically used or designed for paediatrics, so to maximise comparability the McFaul system was used. This rated quality as: 1 – not useful, 2 – partly useful, 3 – moderately good, 4 – very good (safe for clinical practice) and 5 – excellent.

**Table 2:** Acuity scoring grid to assess clinical features seen in video clips (as used by McFaul [9])

Score	0	1	2
Colour	Normal	Pale or Flushed or Mottled	Cyanotic or Ashen
Response to social overture	Chats or smiles OR “alerts” (< 2months)	Single words or briefly smiles OR “alerts” briefly (< 2months)	No smile. Face anxious OR dull and expressionless or no “alertness”
State variation	If awake stays awake OR if asleep and stimulated wakes quickly	Eyes close briefly and then awakens OR awakens after prolonged stimulation	Falls asleep when examined OR will not rouse
Hydration	Skin normal, eyes normal and mucous membranes moist	Skin/eyes normal and mouth slightly dry	Skin doughy or tented and dry mucous membranes and/or sunken eyes
Respiratory effort	No distress	Some distress eg recession	Laboured with grunt or nasal flare OR marked recession OR absent resps

The principal investigator had directly observed the majority of the children in a clinical context as they were videoed, and made a clinical judgement of PVC quality against the McFaul system, which was used as the gold criterion standard. The scenario described to all participants was that they were making a telemedicine judgement based on a video clip shown to them by a member of junior medical staff. They were asked to grade each clip. If they would be comfortable making a clinical decision based on the clip then this would be graded as at least 4 (very good – safe for clinical practice). The participants all viewed the clips from the same angle on the same computer screen (an LCD screen with 1366x768 pixel frame) in the same lighting conditions. No clips were longer than 20 s and the entire time needed per participant to complete all the questions was approximately 25 min. Clip length was chosen pragmatically to clearly demonstrate the clinical sign in question and allow for a manageable amount of cases to be studied in the available time.

Given the novel nature of the methodology power calculations were not undertaken as there were no prior studies with data to enable an estimation of effect size. Stata version 13 was used to analyse data with significance set at *p* < 0.05. Spearman’s rho was used to determine relationships between image quality standards and raters’ quality of assessment. This non-parametric test was chosen as the results were not normally distributed. In order to assess whether the level of significance of the correlation coefficient was affected by the clustering of ratings within the same video clips, corresponding tests were carried out using linear regression with random effects for the clips. Spearman’s rank correlation was used to determine the relationship between gold standard assessment and the raters’ assessment.

## Results

Twelve participants undertook the study. The total number of clips with quality scores was 333 (27 clips in 4 different versions seen by 3 participants at each version and 1 clip seen in 3 different versions by 3 participants at each version). There were three instances where a participant (three different individuals) was unable to make an acuity judgement on the clip. So there were 330 responses with acuity and quality scores for analysis.

There was no relationship between image quality and raters’ quality of assessment (Spearman’s rho = 0.0410, 95 %–0.067 to 0.148, *p* = 0.45) (Table 3). This lack of statistical significance was confirmed in two random effects models one including the repeated clip (*p* = 0.469) and one without it (*p* = 0.677).

**Table 3:** Image quality versus Paediatrician assessment of quality

Quality of image	Paediatrician assessment of quality of image					
	1	2	3	4	5	Total
Low	16	14	17	36	1	84
Medium	9	16	22	30	4	81
High	12	17	25	25	5	84
Excellent	10	11	25	35	3	84
Total	47	58	89	126	13	333

1 Not at all, 2 partly, 3 moderately, 4 Very good [safe for clinical practice], 5 Excellent

Spearman’s rho = 0.0410, 95 % CI: −0.067 to 0.148, *p* = 0.45

There was a better match between the rater’s assessment of acuity and the gold standard (the principle investigator’s clinical assessment) at high acuities (89.9 %) than at the low acuity (67.6 %) (Table 4). The overall correlation was 0.664 based on 330 observations 95 % CI: 0.598 to 0.720.

**Table 4:** Match of the paediatricians’ acuity score with the gold standard

Gold standard severity of patient	Paediatricians’ acuity score (%)			
	1	2	3	Total
1	71 (67.6)	23 (21.9)	11 (10.5)	105
2	16 (10.3)	102 (65.4)	38 (24.3)	156
3	2 (2.90)	5 (7.2)	62 (89.9)	69
Total	89 (27.0)	130 (39.4)	111 (33.6)	330

1 normal clinical findings, 2 moderate derangement, 3 severe illness

The paediatrician’s acuity score versus gold standard varied across the five clinical sign domains. A difference of two levels in either direction (+/− 2) means that the gold standard rated the abnormality as normal or severe and the rater judged the opposite. This is likely to be a clinically significant difference. Social interaction showed the greatest variability with 7 (11.7 %) cases showing a +2 difference and Respiratory showed the least (zero) +2 differences (Table 5).

**Table 5:** Paediatricians’ acuity score versus gold standard across the domains

Clinical sign	Correct	+/− 1 difference	+/− 2 difference	Total
Colour	84 (79.3 %)	20 (18.8 %)	2 (1.9 %)	106
State Variation	45 (75.0 %)	12 (20.0 %)	3 (5.0 %)	60
Response to social overture	42 (70.0 %)	11 (18.3 %)	7 (11.7 %)	60
Hydration	29 (61.7 %)	17 (36.2 %)	1 (2.1 %)	47
Respiratory	35 (61.45)	22 (38.6 %)	0 (0 %)	57
Total	235 (71.2)	82 (24.9)	13 (3.9)	330

The raters’ ability to correctly score acuity showed little variation at the four levels of technical image quality (Table 6). Given there was no previous data to judge the significance of differences in the ratio of correct to incorrect answers no further statistical tests were performed. There was a significant relationship between rater’s perception of quality and the correct score (Spearman’s rho = 0.128; 95 % CI: 0.021 to 0.233; *p* = 0.0196), and this relationship was stronger when the repeated clip was not included (Spearman’s rho = 0.171; 95 % CI: 0.056 to 0.283; *p* = 0.004). The corresponding random effects models were less strongly significant, with *p* = 0.038 and *p* = 0.034 respectively.

**Table 6:** Technical quality of image versus acuity score

Industry defined technical quality of image	Acuity score		
	Incorrect	Correct	Total
Low	22 (26.2 %)	62 (73.8 %)	84
Medium	23 (28.4 %)	58 (71.6 %)	81
High	27 (32.1 %)	57 (67.9 %)	84
Excellent	26 (30.9 %)	58 (69.1 %)	84
Total	98 (29.4 %)	235 (70.6 %)	333

## Discussion

Although quality assurance of videos in respect of descriptive information content has been defined this has not occurred for video image quality [11]. In this study the null hypothesis that the technical quality of a video clip of an acutely unwell child does not influence a paediatrician’s judgement on their acuity was accepted. However the paediatrician’s perception of the quality of the clip did appear to have an effect. It also appeared that judgements between paediatricians were generally cohesive for higher acuity patients but there was greater variation in more mild/moderate illness (i.e., lower acuity illness).

These results imply that the clinicians had a different meaning of “quality” from the technical industry standards. In other words the technical video quality measures do not match the factors which the paediatricians perceived as important in being able to judge acuity in PVCs. Further studies are needed to elucidate the factors underlying a clinician’s perception of video quality. The reasons why the paediatricians may be influenced by their perception of quality rather than the technical quality are complex. Polanyi [12] is credited with coining the term ‘tacit knowledge’, a concept Schön [13] described as knowledge that is usable but which one can not rationally express. Given that all the paediatricians were given the same visual information and, lack of specific clinical details, the intrinsic cognitive load [14] for all was the same. In the absence of a qualitative analysis, it is difficult to know whether experience, clinical knowledge or tacit knowledge contributed to the decisions the consultants made. It may be that the clips rated as poor quality were the cases where there existed the greatest discrepancy between the clinical sign shown and need for further information to evaluate those signs. A young infant with a high respiratory rate and a background of prematurity has a greater risk of subsequent deterioration. This information is normally vital in order to make a decision about care. Although the consultants were not being asked about disposition or treatment they may have struggled making a decision without this type of context.

Context has shown to be very important in decision-making. Croskerry cites examples of system 1 and 2 processing, a psychological theory regarding cognitive reasoning [15]. Sherbino et al. [16] described system 1 as rapid, unconscious, and contextual thinking whereas system 2 is slow, logical, and rational. Kahneman and Tversky [17] put forward the original theory that system 1 thinking results in error when system 2 processes do not spot mistakes during system 1 processing. The consultants when reviewing real patients would be making instinctive judgements, likely with background information readily available or already processed. The insufficient information in this study meaning system 1 decisions were difficult to process and there was not sufficient time in the study to utilise system 2 analysis. Although the system 1 and 2 classification itself has been questioned [18], it is plausible that the quick decisions reached when viewing video footage must be informed by supplementary information. Video assessment may not be authentic without this context because the second phase of cognitive evaluation may not be possible if the initial information is insufficient (clinicians may be concentrating on what is missing rather than what they see).

It is possible that certain clinical signs were more difficult to interpret than others and this may have influenced the relationship between video quality and outcome. There is some evidence for this as there was a greater proportion of agreement with the gold standard for the domains of alertness (75.0 %) and colour (79.3 %) than there was for hydration (61.7 %) or respiratory (61.4 %). This could be considered surprising as colour gradation is affected by the quality of the image and has poor inter-observer reliability even when examined in direct clinical practice [19]. The fact that colour was not commonly incorrectly assigned, low incidence of +1 (18.9 %) and +2 (1.9 %) differences compared to the average +1 (24.9 %) and +2 (3.9 %) difference with gold standard acuity lends credence to the fact that it may not have been the technical quality the participants were judging but an overall information deficit. Ideally a range of acuities in the same patient (or at least similar ages and ethnicities) across a range of qualities could be utilised to try and limit the impact of these potential contextual confounders.

In order to confirm these findings a validation of this study is needed as a number of factors may have influenced the results. The participants may not be representative of typical clinicians as they were all experienced in assessment. It could also be argued that the gold standard was not adequate as less than 70 % of the participants were correct for hydration and respiratory. However, even if the gold standard was accepted to be incorrect, it would still be the case there was large variation in the answers. The purpose of the study was to benchmark quality and observe variability between experts rather than test clinical accuracy, so this pragmatic gold standard was thought to be sufficient. Similarly other measures of video quality could have been varied, such as signal-to-noise ratios, but bitrates were practically the easiest to alter with available resources. However it would be useful to replicate the study using a reference point video as a control to confirm the clinical significance of the bitrates used. A further study altering clip length may also be useful as this may have impacted on the judgement of the paediatrician and it is possible showing the clips for a longer period may have altered the acuity and quality score given.

Preference for a particular cinematic film compared to another has been shown to affect the perceived quality of the video [20]. It is unlikely the paediatricians will have had a favourite video case which may have biased their judgement as these are scenarios they would associate with a need to make a clinical rather than emotional decision. However had an initial study examining the factors influencing clinician’s perception of quality been performed it is possible it may have revealed design features to control for in this research project.

A recent study has demonstrated considerable variability in the assessment of breathing difficulty in wheezy children between professionals [21]. Utilising video to educate and quality assure could become increasingly important and the outcomes of this work may also have application beyond Medical Education as minimum standards would be useful to aid telemedicine service providers to configure data transfer links appropriately.

## Conclusion

It is important to benchmark PVCs prior to use in learning resources as experts may not agree on the information within or quality of clip. Although PVCs may be beneficial in a pedagogical context their translation into an assessment methodology or clinical use via telemedicine must be further examined as perception of quality of clip was an important determinant of an expert’s decision-making.
